# Paper battery powered iontophoresis microneedles patch for hypertrophic scar treatment

**DOI:** 10.1038/s41378-024-00823-0

**Published:** 2025-03-10

**Authors:** Jie Gao, Fuqian Chen, Chen Wang, Jingbo Yang, Ying Zheng, Bin Liu, Gang Nie, Linyu Zhu, Shuo Wu, Xi Xie, Lelun Jiang

**Affiliations:** 1 Guangdong Provincial Key Laboratory of Sensor Technology and Biomedical Instrument, School of Biomedical Engineering, Shenzhen Campus of Sun Yat-Sen University, Shenzhen, 518107 PR China; 2 Department of Dermatovenereology, The Seventh Affiliated Hospital, Sun Yat-sen University, Shenzhen, 518107 PR China; 3 Department of Otolaryngology, The Third Affiliated Hospital, Sun Yat-Sen University, Guangzhou, 510630 PR China; 4 State Key Laboratory of Optoelectronic Materials and Technologies, School of Electronics and Information Technology, Sun Yat-sen University, Guangzhou, 510006 PR China

**Keywords:** Electrical and electronic engineering, Chemistry

## Abstract

Hypertrophic scar (HS) is a plaque fibrous and indurated dermal lesion that may cause physical, psychological, and cosmetic challenges for patients. Intralesional injection of triamcinolone acetonide (TA) is commonly used in clinical practice, which cause unbearable pain and uneven drug delivery within HS tissue. Herein, we developed a paper battery powered iontophoresis-driven microneedles patch (PBIMNP) for self-management of HS. The high integration of PBIMNP was achieved by incorporating a paper battery as the power source for iontophoresis. The transdermal drug delivery strategy of PBIMNP combined microneedles and iontophoresis techniques, involving “pressing and poking, phase transformation, and diffusion and iontophoresis”, which can actively deliver 90.19% drug into the HS tissue with excellent in vitro drug permeation performance. PBIMNP administration effectively reduced the mRNA and protein levels, leading to a decrease in the expression of TGF-*β*1 and Col I associated with HS formation, demonstrating its efficacy in HS treatment. The microneedles and wearable design endow the PBIMNP as a highly promising platform for self-administration on HS treatment.

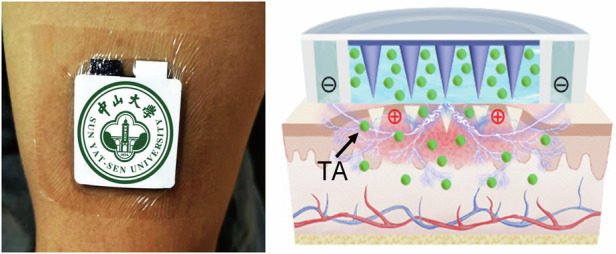

## Introduction

Hypertrophic scar (HS) is characterized by excessive fibroblast proliferation and collagen deposition resulting from abnormal wound healing following skin injury such as surgery, burns, scalds, or body perforation^1,2^. It leads to reduced tissue permeability due to increased tissue thickness^3,4^, presenting as red, thickened, hard skin plaques on dermoscopy. Patients may experience pain, pruritus, dysfunction and other issues associated with physical, psychological and cosmetic concerns^5,6^. The treatments for HS include surgery, radiotherapy, laser treatment, pressure therapy, and intralesional injection^7^, which have certain limitations, such as a high recurrence rate after surgery or laser treatment, serious side effects of radiotherapy, and a long treatment cycle of pressure therapy^8,9^. The injection is the primary therapeutic approach for HS treatment^10^. The primary clinical intervention involves the intralesional administration of glucocorticoids, with triamcinolone acetonide (TA) being the most commonly employed glucocorticoid since 1960^11,12^. The intense pain caused by the high pressure during injection administration is unbearable for most patients, even with anesthesia, leading to poor patient compliance^13^. Moreover, point injection often leads to uneven distribution of delivered drug, giving rise to adverse effects, such as skin atrophy, pigment loss and menstrual disorders^14^.

To address the challenges of pain and uneven drug delivery in clinical injection for HS treatment, microneedles (MN) have been regarded as a promising strategy for transdermal drug delivery due to their unique advantages, including minimal skin trauma, self-administration, and ease of disposal^15^. However, the use of drug-free MN for HS treatment has shown limited therapeutic effects and necessitated long-term wearing (30 days)^16,17^. Incorporating clinical drugs into dissolvable MN effectively was employed to address the challenges of intense pain and inconsistent drug delivery in HS treatment using point injection. Nevertheless, the complex fabrication process and poor mechanical properties pose difficulties in puncturing dense and hard HS tissue^13,18,19^.The size and performance of microneedles patches has shown in Table S1. Compared with clinical intralesional injection, MN-based delivery approaches for HS treatment typically rely on relatively slow passive diffusion of drugs through microchannels created by MN insertion or dissolution of drug-loaded MN^20^.

Iontophoresis technique also has recently been integrated with wearable devices to electrically control and enhance the permeation of small hydrophilic drug molecules across the skin for HS treatment. The transdermal drug delivery of iontophoresis technique is achieved using a mild current through electrophoresis and electroosmotic flow^21–23^. Iontophoresis effectively enhances drug delivery in HS, as demonstrated by the significant increase in sodium fluorescein delivery and improvement of morning stiffness scores with acetic acid iontophoresis^24,25^. The main advantage of iontophoresis is the precise control over the electrified time and parameters, enabling accurate delivery. However, the iontophoresis technique is excellent for delivering small molecules, but it faces challenges in delivering macromolecules due to the barriers of the SC. Moreover, the iontophoresis usually require large external power supply, which were inconvenient for use^26,27^. To overcome these limitations, the integration of MN with iontophoresis techniques in a wearable drug delivery patch holds great potential to synergistically enhance the efficacy of drug administration for HS treatment.

**Fig. 1:**
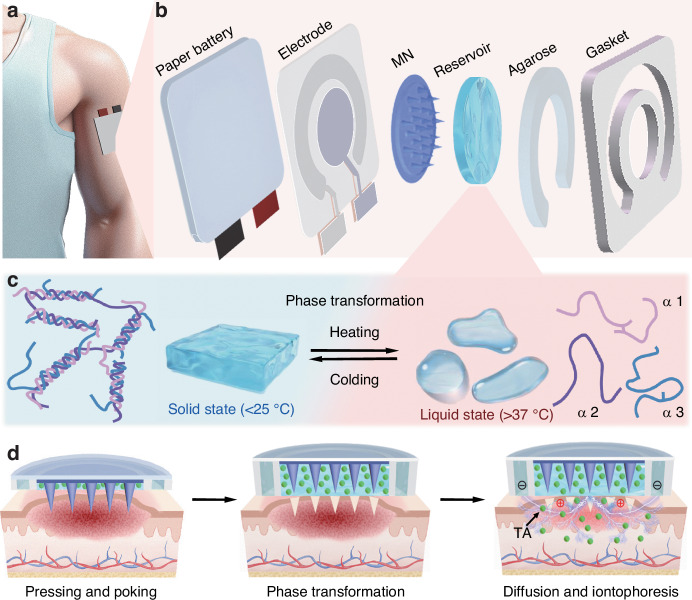
Schematic illustration of PBIMNP. **a** Schematic diagram of PBIMNP. **b** Explosion view of PBIMNP. **c** The solid-liquid transformation mechanism of gelatin. The pink (*α*1), purple (*α*2), and blue (*α*3) lines represent the three branches of the gelatin triple helix structure. **d** The drug delivery strategy of PBIMNP: pressing and poking, phase transformation, diffusion and iontophoresis. The green dots represent TA drugs

Herein, a paper battery powered iontophoresis-driven microneedles patch (PBIMNP) was proposed for HS self-administration and personalized treatment, as shown in Fig. 1. The drug delivery strategy of PBIMNP involves “pressing and poking, phase transformation, and diffusion and iontophoresis”, in which the MN served as a tool for penetrating HS in a pain-free manner to create microchannels, and then the TA drugs are delivered through these microchannels via passive diffusion and active iontophoresis in an electrically controlled manner. The power supply of iontophoresis was facilitated by a thin paper battery, eliminating the necessity for the large external power supply and effectively minimizing the size of the wearable PBIMNP device. The solid metal MN owns good mechanical properties, enabling effective penetration through the dense and rigid HS tissue. PBIMNP can realize the easy storage and controllable release of hydrophobic drugs through the phase transformation of the gelatin. The implementation of PBIMNP may promote self-care and personalized treatment of HS, providing a promising alternative to the traditional methods of clinical.

## Results and discussion

### Design of the PBIMNP

The PBIMNP was developed for HS self-management and personalized treatment, as shown in Fig. 1a. PBIMNP mainly consists of three modules (as shown in Fig. 1b): (1) Drug storage module: an impermeable ring, an agarose gel, and a gelatin. The gelatin is loaded with TA. The impermeable ring separates the anode and cathode for iontophoresis and provides resilience to MN. The agarose gel and gelatin are served as the anode and cathode of iontophoresis, respectively. (2) MN module: The MN is employed to puncture the thickened corneum and create the microchannels for drug diffusion. (3) Iontophoresis module: the paper battery connected with the flexible PCB provides a consistent current for iontophoresis. When PBIMNP is adhered on the HS skin, the solid gelatin loaded with TA is gradually transformed into a liquid state due to the heat transfer from the skin. Then the iontophoresis-driven drug delivery is triggered due to the formation of a closed conductive loop between the reservoir and the agarose gel through skin. The paper battery and the flexible PCB for iontophoresis circuit is extremely simple, which makes the PBIMNP highly integrated. Moreover, the exceptional flexibility of the paper battery and PCB allows the PBIMNP closely conform to the skin, contributing to its outstanding adaptability. The reservoir exhibits a temperature-sensitive property, enabling efficient storage and controlled release of TA at physiological temperature (37 °C).

The drug delivery strategy of PBIMNP involves “pressing and poking, phase transformation, and diffusion and iontophoresis”, as illustrated in Fig. 1d. (1) Pressing and poking: the PBIMNP is conformably attached to the HS skin. The MN tips penetrate through the reservoir and HS tissue by applying a pressure on the PBIMNP, and the MN detach from the HS upon removal of the compressed, thereby creating microchannels for transdermal drug delivery. (2) Phase transformation: gelatin is a thermoreversible gel, and its phase transformation temperature (Tt) is close to the human physiological temperature^28,29^. Figure 1c shows the phase transformation of the gelation. Specifically, when gelatin is cooled below room temperature, the peptide chains gather and form a triple helix structure, thus creating a three-dimensional network gel^30,31^. When the temperature exceeds Tt, the gel undergoes a reverse phase transformation and transform into a liquid state^32,33^. Upon the application of PBIMNP to HS tissue, the reservoir undergoes a phase transformation triggered by physiological temperature and subsequently release the drug for transdermal delivery. The phase transformation duration is short within 2 to 3 min. (3) Diffusion and iontophoresis: the drug in reservoir passively diffuses into the HS skin via the poked microchannels created by MN along the concentration gradient according to Fick’s diffusion law. The rate of passive diffusion is mainly determined by the concentration of TA in reservoir and the created microchannels. Meanwhile, the iontophoresis current between PBIMNP and HS skin actively drives the drug to permeate into HS tissue through the pocked microchannels due to the electroosmosis and electromigration. The iontophoresis facilitates solvent convection, thereby driving the drug into the HS tissue through electroosmosis. Additionally, the repulsive force on the reservoir enhances TA migration to the HS tissue. The majority of the electroosmotic flow during iontophoresis migrates along low-resistance and preferred pathways, primarily associated with the microchannels^34^. The iontophoresis-driven delivery rate is primarily determined by the drug concentration, the microchannels created by the MN, and the current and duration of iontophoresis. Moreover, the combination of passive diffusion and active iontophoresis-driven penetration may synergistically enhance the efficacy of transdermal drug delivery.

### Characterization of PBIMNP

**Fig. 2:**
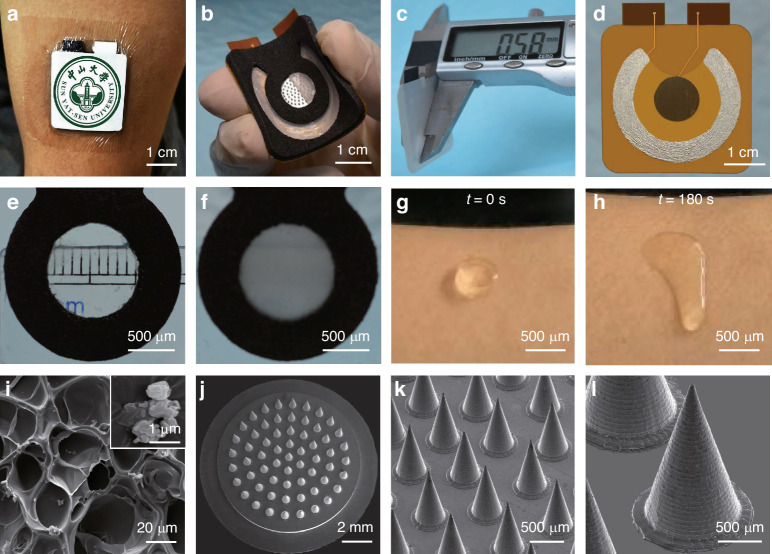
Characterization of PBIMNP. **a** The image of PBIMNP fixed on the upper arm. **b** The image of flexible PBIMNP. **c** The thickness of the paper battery. **d** The iontophoresis-driven circuit on a flexible PCB. **e** The optical image of gelatin at approximately 25 °C. **f** The gelatin wrapped with TA. **g** The solid gelatin on the skin at *t* = 0 s. **h** The liquid gelatin due to the phase transformation at *t* = 180 s. **i** The SEM image of gelatin wrapped with TA. Insert: The SEM image of TA powders. **j**–**l** The SEM images of MN

An assembled PBIMNP is illustrated in Fig. 2a. Figure S1 shows the design of the PBIMNP. Video S1 presents the assembly process of PBIMNP. The size of PBIMNP is 35 × 40 × 2.1 *mm*^3^, and the weigh is only 2.63 g, exhibiting good flexibility and thereby ensuring a high conformation on the skin (Fig. 2b). The patch can smoothly adhere to the skin without drug leakage. Moreover, the paper battery for iontophoresis is incredibly thin, measuring only 0.58 mm in thickness and weighing around 1 g, easily bending with a minimum radius of 30 mm (Fig. 2c). The iontophoresis-driven circuit was designed on a flexible PCB. The cathode was coated with Ag/AgCl ink, as depicted in Fig. 2d. The gelatin block demonstrated transparency under room temperature of approximately 25 °C (Fig. 2e). The reservoir was prepared by uniformly incorporating TA powder into the gelatin (Fig. 2f). The reservoir exhibits a uniformly opaque appearance attributed to the homogeneous dispersion of TA powder within the gelatin. Additionally, the gelatin was applied onto the forearm to observe its phase transformation at physiological temperature, as shown in Fig. S2 and Video S2. Figure 2g, h present the gelatin and phase transformation-gelatin, respectively. The conductivity of gelatin is approximately 0.0436 S/m. Fig. 2g demonstrates that the gelatin can effectively conform to the skin, ensuring good conductivity between the electrode and the skin. The gelatin can be transformed from solid gel into liquid within 180 s, exhibiting good fluidity. The SEM image of gelatin exhibits a uniform distribution of pores, suggesting its potential for efficient drug storage (Fig. S4a). Figure 2i shows the SEM of gelatin-wrapped TA. TA powders are evenly dispersed in the pores of the gelatin. Figure S3 shows the characterization of TA. The average diameter of TA powers is less than 187.5 *μ*m (Fig. S3a). The zeta potential of TA particles was 3.76 ± 0.25 *mV* (Fig. S3b). The MN should possess outstanding mechanical properties and sharp tips, thereby enabling effective penetration of dense HS tissue without breakage. The MN morphology is shown in Figs. 2j–l and S4. It uses a two-stage roundtable design with an inner diameter of 11 mm and an outer diameter of 13 mm, which can be exactly equipped with the impermeable ring to prevent drug leakage. The MN consists of 61 conical microneedles, uniformly arranged on the inner roundtable. The average height, tip diameter, and base diameter of conical microneedles were approximately 1000 *μ*m, 20 *μ*m, and 600 *μ*m, respectively. The conical microneedles with sharp tips may guarantee the skin penetration. The spiral grain can be observed on the surface of conical microneedle, which is attributed to the fabrication process of micro milling (Fig. 2l).

### Performance of PBIMNP

**Fig. 3:**
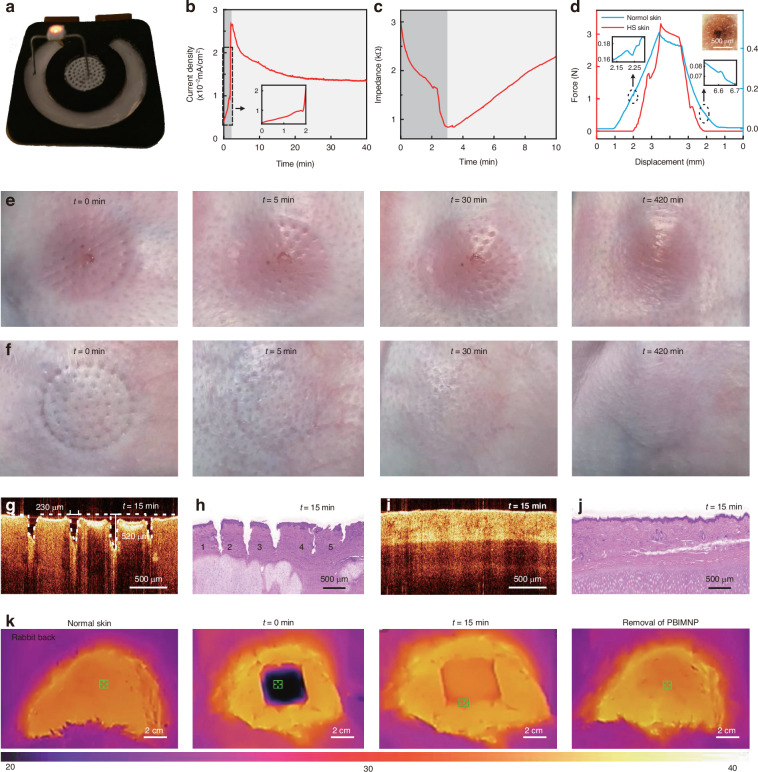
The basic performance of PBIMNP. **a** A LED is lightened by PBIMNP. **b** The current density of PBIMNP during drug delivery. **c** Impedance performance of PBIMNP during drug delivery. **d** The penetration performance of PBIMNP on HS. **e** HS skin recovery after puncture. **f** Normal skin recovery after puncture. **g** OCT image of the microchannels in HS skin recovery at 15 min after puncture. **h** HE image of the microchannels in HS skin recovery at 15 min after puncture. **i** OCT image of the microchannels in normal skin recovery at 15 min after puncture. **j** HE image of the microchannels in normal skin recovery at 15 min after puncture. **k** Heat mapping on the back of a New Zealand rabbit treated with PBIMNP at 0 min, 1 min, 15 min and removal of PBIMNP

The PBIMNP can light up a 1.5 V LED using the iontophoresis circuit (Fig. 3a), demonstrating the good conductive of the PBIMNP. The paper battery can stably output a voltage of approximately 1.4 V when subjected to both a constant resistance of 2000 *Ω* and varying resistances ranging from 500 to 3000 *Ω* (Fig. S5), indicating that the paper battery can provide a reliable power supply for PBIMNP, ensuring the stable drug delivery. Figure 3b shows the current density of PBIMNP during iontophoresis-driven drug delivery. The PBIMNP forms a circuit with the skin, resulting in a gradual increase in current density. The gelatin undergoes phase transformation from solid to liquid within approximately 2 min due to heat conduction from human body. The phase transformation increases skin humidity and reduces impedance, thereby instantaneously increases the current. Subsequently, the microchannels created by MN puncture allow for both passive diffusion and active iontophoresis, facilitating effective drug delivery into HS tissue. The current density gradually decreased over time and eventually reached a stable state after approximately 30 min. The crucial step encompasses phase transformation, passive diffusion and active iontophoresis-driven penetration. Figure 3c shows the impedance curve during drug delivery of PBIMNP. The impedance decreases at first 2 min due to the phase transformation of gelatin and then gradually increases owing to the decrease of skin humidity during drug delivery. The long-term stability gelatin loaded with TA stored at 4 °C for 28 days was tested (Fig. S6). The pharmaceutical effect remained at 90% after 28 days, indicating high stability of TA in the gelatin.

The HS tissue has a high density and hardness, making it difficult to be punctured^16^. The penetration performance of PBIMNP on HS and normal skin was investigated (Fig. 3d). During the pressing and poking stage, the resistance force increased as the PBIMNP was compressed. Once the resistance force reaches the rupture limit, the MN pierces into the skin, resulting in an abrupt decrease in force at point “P”. The critical penetration force of HS skin was 1.7 N/needle, which was 10 times higher than the force for normal skin (0.17 N/needle). It was further demonstrating that the low-tension strength of HS tissue, requiring a higher force to achieve effective puncture of PBIMNP. During the release stage, the measured force gradually decreases upon gradual removal of compression on the PBIMNP. Once MN is detached from the skin, the friction decreases to 0, corresponding to an increase at point “Q” (Fig. 3d). Figure S7a, b show OCT and HE images of HS tissue after puncture with PBIMNP. The average depth and base width of the poked microchannel were 600 and 300 *μ*m, respectively. The mechanical failure performance of PBIMNP poking on steel plate was also investigated (Fig. S7c). The resistance force gradually increases with load displacement. The failure force reached 13.1 N/needle, which was much higher than the penetration force of HS tissue (1.7 N/needle), demonstrating MN owns enough mechanical strengthen for skin penetration without damage. After poking the plate, microneedles were bent without broken (insert in Fig. S7c), guaranteeing the safety of MN without broken in the skin, thereby effectively preventing foreign body and inflammatory reactions.

Figure 3e, f show the recovery of microchannels after PBIMNP puncture in HS skin and normal skin on rabbit in vivo, respectively. Figure S8 shows the recovery process of microchannel in HS and normal skin over 420 min. The microchannels in HS skin punctured by PBIMNP still can be observed after 30 min, and completely healed within 420 min. However, the microchannels in normal skin was almost heal within 5 min. No obvious signs of erythema and lesions were observed on the skin surface after recovery. The slow recovery of microchannels in HS tissue may be attributed to its skin tension^35^. Due to the high tension of normal skin in rabbit ears, the microchannels were rapidly healed within 5 min, much faster than the mouse skin (1 h)^36^. The persistence of microchannels in the skin may facilitate drug delivery to deep tissues through iontophoresis and diffusion, thereby enhancing therapeutic efficacy^37^. To evaluate the penetration depth of the drug in the skin, the skin stained with rhodamine B was observed by laser confocal microscopy, as shown in Fig. S9. Compared with the passive diffusion, MNP and PBIMNP can increase drug diffusion depth in the skin. Among them, the PBIMNP group showed the most significant effect, indicating its higher potential to promote the deep diffusion of drugs. The punctured microchannels in HS and normal skin after 15 min recovery was further investigated, as shown in Fig. 3g–j. The depth and diameter of poked microchannels in HS skin were 520 *μ*m and 230 *μ*m (Fig. S7a, initial microchannel depth of 600 *μ*m and diameter of 300 *μ*m), respectively, indicating slow healing of the microchannels. Comparably, the microchannels in normal skin were completely healed at 15 min. As shown in Fig. 3h, the deposition of extracellular matrix (ECM) was found to be abundant around microchannels “1–3”, indicating a more serious scar differentiation, whereas the deposition of ECM was comparatively lower around microchannels “4–5”, suggesting an accelerated scar recovery. Due to the presence of cartilage, the microchannels in normal skin healed completely after puncture recovery at 15 min (Fig. 3j). The excessive deposition of ECM in HS tissues leads to a decrease in skin tension, resulting in prolonged microchannel healing time and thereby enabling the longer-lasting drug diffusion and iontophoresis^16^.

The iontophoresis current may generate heat energy and thereby burns the skin, so the temperature distribution of PBIMNP on the rabbit back during drug delivery was observed using an infrared camera (Fig. 3k). Video S3 shows the thermal map of PBIMNP upon application to the rabbit dorsal skin for 30 min. The initial temperature of PBIMNP was at 4 °C as it had been stored in a refrigerator. Upon application of PBIMNP onto the skin, the skin temperature rapidly decreased and the average temperature of PBIMNP gradually increased to 34.7 °C within 15 min due to heat conduction from the skin. Subsequently, the PBIMNP temperature remained stable at 35.5 °C without any further increase, indicating minimal influence of iontophoresis current on thermal damage to the skin.

### Cell proliferation and toxicity of PBIMNP on HSFBs

**Fig. 4:**
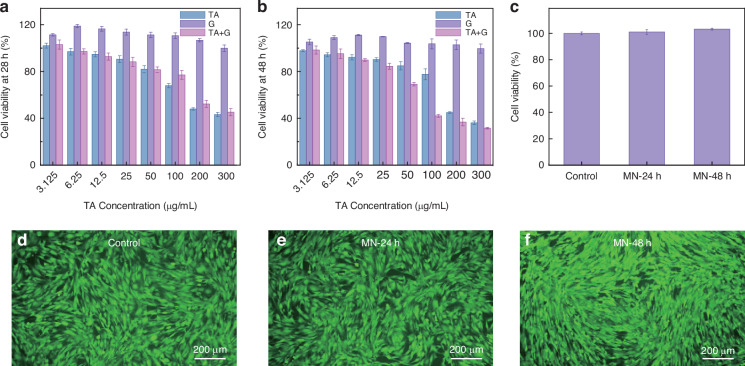
Cell proliferation and toxicity of PBIMNP on HSFBs. Cell viability of HSFBs treated with TA, gelatin and gelatin-wrapped TA at 24 h (**a**) and 48 h (**b**) (*n* = 3). The G represents the gelatin. **c** Cell viability of HSFBs incubated with MN for 24 h and 48 h. Fluorescence images of HSFBs stained by live/dead assay in control (**d**), MN at 24 h (**e**) and MN at 48 h (**h**), where live and dead cells show green and red fluorescence, respectively

The gelatin load with TA in PBIMNP is delivered into HS tissue via a combination of passive diffusion and iontophoresis. Collagen-derived gelatin exhibits low immunogenicity, high biodegradability, and excellent biocompatibility^38^. The TA administration can attenuate inflammation, leading to a reduction in the synthesis of collagen and glycosaminoglycan, subsequently resulting in the degradation of collagen and fibroblast^39^. Moreover, we evaluated the inhibition effects of TA, gelatin, and gelatin-wrapped TA on HSFBs. As shown in Fig. 4a, b, HSFBs (human hypertrophic scar fibroblasts) exhibited a concentration-dependent response to TA concentration. Moreover, there was no significant difference in cell viability between 24 h and 48 h, indicating that the inhibit effect of TA on HSFBs was concentration-dependent. The gelatin at different concentrations promoted the proliferation of HSFBs due to its good biocompatibility. Additionally, we assessed the biocompatibility of MN (Fig. 4c). The viability of HSFBs remained above 100% at 24 and 48 h, demonstrating good biocompatibility. The cytotoxicity of MN was also evaluated using AM/PI kit (Fig. 4d–f). Compared with the control group, the number of dead cells after incubation for 24 and 48 h did not show any increase, further confirming the excellent biocompatibility of MN.

### In vitro permeation of PBIMNP in HS skin

**Fig. 5:**
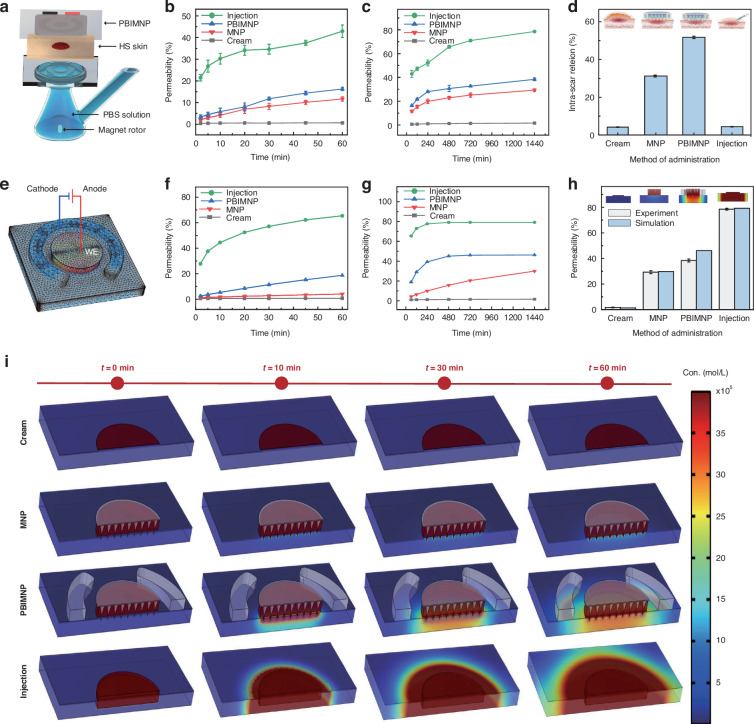
In vitro transdermal delivery performance and finite element analysis of PBIMNP. **a** Schematic diagram of in vitro transdermal drug delivery of PBIMNP using Franz diffusion cells. **b** In vitro permeability of the four administration approaches at 60 min. **c** In vitro permeability of the four administration approaches at 1440 min. **d** The intra-scar retention in HS after in vitro permeability tests. **e** Schematic illustration of the FEA model of PBIMNP. **f** The calculated cumulative amount administrated with different methods at 60 min. **g** The calculated cumulative amount administrated with different methods at 1440 min. **h** Comparison of the final cumulative amount at 24 h between the in vitro experimental and calculated groups (data are mean ± SD, *n* = 3). **i** The calculated transdermal delivery process administrated with different methods

To explore the transdermal delivery performance of the PBIMNP on HS skin, vertical Franz diffusion cells were custom-designed for in vitro permeation tests, as shown Fig. 5a. The in vitro permeability of Cream, MNP, PBIMNP and Injection groups was evaluated (Fig. 5b–d). Figure 5b, c showed the in vitro permeability of Cream, MNP, PBIMNP and Injection groups for 60 min and 1440 min, respectively. It was found that only 1.65 ± 0.55% of TA permeated through the HS tissue using Cream at 1440 min, while the MNP, PBIMNP and Injection groups showed significantly improved permeability of 29.32 ± 1.96%, 38.41 ± 2.04%, and 78.56 ± 1.31%, respectively. Due to the delivery microchannels poked by MN, the permeability of MNP and PBIMNP groups significantly increased, which were 17.77 and 23.27 times of Cream group, respectively. Compared with MNP, PBIMNP showed superior permeability in HS skin, which was attributed to the iontophoresis, enhancing its permeability 1.31 times higher than that of MNP group. Figure 5d shows the retention of TA in HS skin following in vitro permeation tests. The drug retention rates of Injection and Cream groups were only 4.41% and 4.2% in HS skin at 1440 min, respectively. However, PBIMNP exhibited excellent retention ability with a drug retention rate of 51.78% at 1440 min, indicating its positive significance for treating HS skin. PBIMNP effectively delivered 90.19% of the drug to the HS tissue (38.41% penetrated into the Franz diffusion cells, 51.78% remained in the HS tissue), demonstrating a remarkably efficient drug delivery.

Furthermore, the transdermal drug delivery mechanism administrated with Cream, MNP, PBIMNP and Injection were analyzed using the FEA method. The 3D drug delivery model was established using COMSOL Multiphysics, utilizing the physical fields of diluted species interface and voltage coupling, as illustrated in Fig. 5e. The permeability of four administration approaches were further calculated based on the simulation, as shown in Fig. 5f, g. The permeability rates of Cream, MNP, PBIMNP, and Injection at 1440 min were determined to be 1.36%, 29.83%, 46.12%, and 79.26% respectively. Notably, the calculated permeability exhibited a highly consistent trend with the in vitro experimental results (Fig. 5h). Figure 5i shows the drug concentration distribution of four administration approaches during the permeation. Injection group directly injected the drug into HS skin and effectively avoid the barrier effect of skin, so the permeability was the highest. However, there was no significant increase in permeability observed within 60 min for Cream group owing to the thick corneum and dense structure of HS skin. The permeability of MNP gradually increased due to the poked microchannels in HS skin by MN. Notably, PBIMNP exhibited a 1.55-fold higher permeability compared to MNP, which was consistent with our earlier in vitro findings. Figure S10 shows the flow field distribution of drug under four different administration. Compared with the passive diffusion, MNP and PBIMNP can increase drug diffusion depth in the skin. Among them, the PBIMNP group showed the most significant effect, indicating its higher potential to promote the deep diffusion of drugs. PBIMNP further enhanced the drug permeability and increased the delivery depth in the HS skin by introduction of iontophoresis with MN, demonstrating the effectiveness and controllability of active iontophoresis on HS treatment.

### In vivo permeation of PBIMNP in HS tissues

**Fig. 6:**
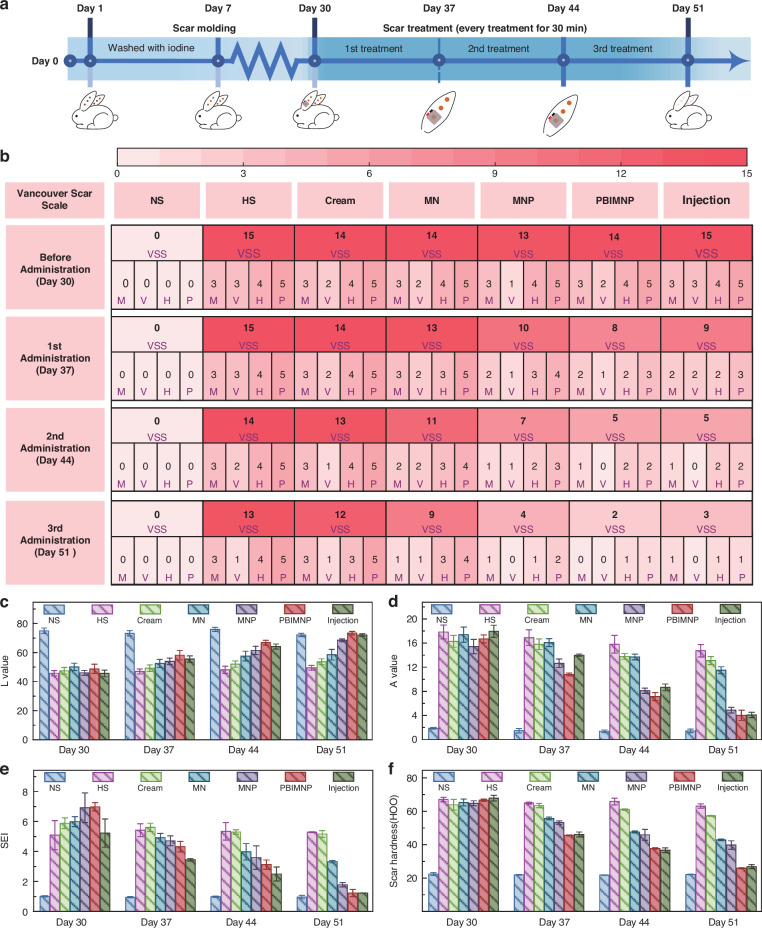
In vivo drug administration performance of PBIMNP. **a** Time point of drug administration process of PBIMNP on rabbit ear scar. **b** Heat map of VSS before and after administration. The M, V, H, and P represent the color (M), vascular distribution (V), thickness (H) and softness (P), respectively. **c** Brightness of HS skin before and after administration. The L represents the brightness of the scar. The higher of L value, the whiter of the brightness. **d** Color of HS skin before and after administration. The A represents the redness of the scar. The higher of A value, the redder of the color. **e** SEI changes of HS skin before and after administration. **f** Hardness changes of HS skin before and after administration

Figure 6a shows the time points of the in vivo administration experiment in rabbit ear scars. The entire experiment lasted for 51 days, including 30 days for HS formation and 21 days for HS treatment. The ear scars were treated 3 times for 30 min, and then the treatment efficacy was evaluated using the Vancouver Scar Scale (VSS) after 7 days. The VSS evaluation of HS tissue was based on four aspects: color (M), vascular distribution (V), thickness (H) and softness (P). The VSS scale ranges from 0 to 15, with higher scores indicating a greater scars severity. Figure 6b shows the heat map of VSS score before and after ear scars treatment. The VSS score of PBIMNP administration is 2 on day 51, slightly lower than that of Injection (a VSS score of 3), indicating a favorable therapeutic outcome. However, it should be noted that the Injection administration involves pain and requires skilled professionals. Additionally, the treatment effect can be visually observed through scar morphological changes during PBIMNP administration (Fig. S11). The recovery of ear scars was assessed before and after treatment, with a comprehensive evaluation conducted on four aspects: brightness, color, Scar Elevation Index (SEI), and hardness, as shown in Fig. 6c–f. The PBIMNP group exhibited the most remarkable therapeutic effect after 3 cycles of treatment, with skin brightness approaching normal levels. Although the color of the PBIMNP group did not reached the normal state, a significant improvement was observed compared to pretreatment. Furthermore, there were significant improvements in both the SEI and hardness of HS skin.

### Histological study

**Fig. 7:**
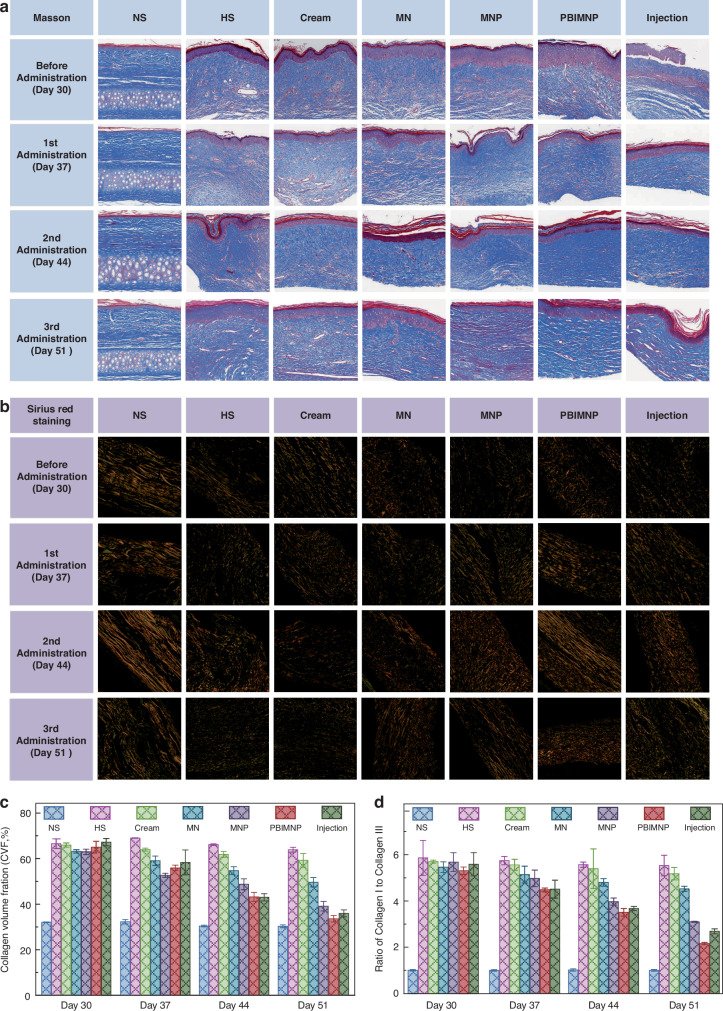
Molecular level of HS tissue before and after various administration. **a** Relative mRNA expression of TGF-*β*1 in HS tissues before and after treatment. **b** Relative mRNA expression of Col I in HS tissues before and after treatment. **c** Protein expression of TGF-*β*1 and Col I in HS tissues. **d** Relative protein expression of TGF-*β*1 in HS tissues before and after treatment. **e** Relative protein expression of Col I in HS tissues before and after treatment. *p < 0.05 vs. NS; **p < 0.01 vs. NS; ***p < 0.001 vs. NS; ^*#*^*p* < 0.05 vs. HS; ^*##*^*p* < 0.01 vs. HS; ^*###*^*p* < 0.001 vs. HS; ^*$*^*p* < 0.05 vs. the same group of the previous week; ^*$$*^*p* < 0.01 vs. the same group of the previous week; ^*$$$*^*p* < 0.001 vs. the same group of the previous week

The collagen in HS tissue was Masson stained and its content could be evaluated using the collagen volume fraction (CVF), as shown in Fig. 7a–c. Compared with other administration approaches, the distribution of collagen fibers administrated with PBIMNP on day 51 presented a regular and loose arrangement, close to normal tissue. The HS tissue exhibited a high density of collagen fibers arranged in a disorganized manner before treatment (on day 30). Following 1st administration (on day 37), some collagen fibers of PBIMNP group showed improved uniformity. Subsequent to 2nd administration (on day 44), although significant deposits were still present, more uniformly aligned collagen fibers were observed in the PBIMNP group. Finally, after 3rd administration (on day 51), evenly distributed and loosely arranged collagen fibers were evident in the PBIMNP group. After 3rd administrations, no significant difference was found in the distribution of collagen fibers between the PBIMNP group and normal tissue. The changes of collagen content were further quantified using CVF, as shown in Fig. 7c. The CVF of the PBIMNP group on day 51 was 33.6 ± 1.41%, slightly lower than that of Injection group (35.97 ± 1.53%), and comparable to the normal tissue (30.29 ± 0.62%). It indicates that the PBIMNP can achieve a better therapeutic effect than typical injection. In addition, we studied the somatic cell count during HS treatment, as shown in Fig. S12a, b. The somatic cell count in HS tissue of PBIMNP group was 1666 ± 23/*mm*^3^ on day 30 and 838 ± 30/*mm*^3^ on day 51, and the normal tissue was 786 ± 7.94/*mm*^3^, indicating a statistically significant reduction. Previous studies have demonstrated that the ratio of type I to type III collagen fibers in normal skin is approximately 1:116, while an imbalance in this ratio was observed in the HS. Figure 7b shows the Sirius Red sections under polarized light before and after HS treatment, while Fig. 7d shows the ratio of type I and type III collagen fibers in Sirius Red sections. The ratio of type I and type III collagen fibers of PBIMNP group was 2.16 ± 0.05 on day 51, slightly lower than that of Injection group (2.68 ± 0.1), but higher than that of normal group (1 ± 0.04). The finding demonstrates a significant improvement compared to the untreated HS group (5.31 ± 0.16), indicating that PBIMNP effectively enhances both the distribution and composition of collagen fibers in HS tissues, thereby holding crucial clinical significance.

### Molecular level research

**Fig. 8:**
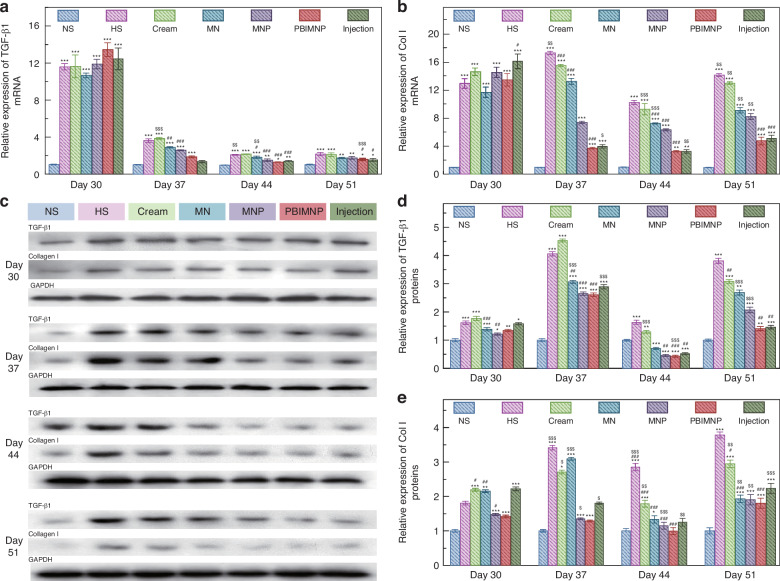
Molecular level of HS tissue before and after various administration. **a** Relative mRNA expression of TGF-*β*1 in HS tissues before and after treatment. **b** Relative mRNA expression of Col I in HS tissues before and after treatment. **c** Protein expression of TGF-*β*1 and Col I in HS tissues. **d** Relative protein expression of TGF-*β*1 in HS tissues before and after treatment. **e** Relative protein expression of Col I in HS tissues before and after treatment. *p < 0.05 vs. NS; **p < 0.01 vs. NS; ***p < 0.001 vs. NS; ^*#*^*p* < 0.05 vs. HS; ^*##*^*p* < 0.01 vs. HS; ^*###*^*p* < 0.001 vs. HS; ^*$*^*p* < 0.05 vs. the same group of the previous week; ^*$$*^*p* < 0.01 vs. the same group of the previous week; ^*$$$*^*p* < 0.001 vs. the same group of the previous week

The over expression of TGF-*β*1 and Collagen I (Col I) is closely associated with HS formation^18^. The analysis of the mRNA and protein expressions of TGF-*β*1 and Col I can further explain the recovery mechanism of HS^13,40^. Figure 8a shows the mRNA relative expression of TGF-*β*1 before and after HS treatment. The mRNA expression of TGF-*β*1 in the PBIMNP group significantly increased on 30 day, which was 11.93 ± 0.94 times higher than that of normal skin. After 3rd administration, the expression of TGF-*β*1 in HS tissue administrated with PBIMNP was significantly decreased to 1.59 ± 0.12 times that of the normal tissue. Figure 8b shows the mRNA relative expression of Col I before and after HS treatment. The expression of Col I in PBIMNP group was reduced to only 4.94 ± 0.55 times that of normal skin. It demonstrates that PBIMNP administration can effectively increase the mRNA expression of TGF-*β*1 and Col I, achieving successful HS therapy.

Figure 8c shows the protein expression of TGF-*β*1 and Col I in HS tissues measured by western blotting (WB). Prior to administration, all groups showed high expression of both proteins (almost 1.5–2.5 times of normal tissues). Figure 8d, e shows the gray scale of TGF-*β*1 and Col I, respectively. The expression of TGF-*β*1 and Col I in the PBIMNP group was 1.69 ± 0.11 times and 1.88 ± 0.37 times than that of normal tissues on day 30, respectively. After 3rd administration, compared to other groups, the protein expression of TGF-*β*1 and Col I significantly decreased in PBIMNP administration. It demonstrates that PBIMNP can significantly reduce the protein expression of TGF-*β*1 and Col I, further confirming the effectiveness of PBIMNP in HS treatment.

## Methods

### Materials and animals

Triamcinolone acetonide (TA, Purity: 99%, relative molecular weight: 434.5 g/mol) was purchased from Shanghai Macklin Biochemical Co., Ltd (Shanghai, China). Gelatin (gum strength of 100g Bloom) was purchased from Shanghai Aladdin Biochemical Technology Co., Ltd (Shanghai, China). Human hypertrophic scar fibroblasts (HSFBs) was obtained from Shanghai Qiansi Biotechnology Center (Shanghai, China). Dulbecco’s Modified Eagle Medium:Nutrient Mixture F-12 (DMEM/F-12) (Gibco), fetal bovine serum (FBS) (Gibco), 0.25% Trypsin-EDTA (Gibco), and Penicillin - streptomycin (10,000 U/mL) were purchased from Thermo Fisher Scientific Co., Ltd (Massachusetts, USA). Cell Counting Kit-8 (CCK-8) was got from Dojindo Molecular Technologies, Inc (Kyushu, Japan). Rabbit Anti-GAPDH (bs-2188R) and Rabbit Anti-TGF beta 1 antibody (bs-0086R) were purchased from Beijing Bioss Antibodies Biotechnology Co., Ltd (Beijing, China). Anti-collagen I Rabbit pAb (WL0088) was provided by Wanleibio Co., Ltd (Liaoning, China). BCA protein detection kit was purchased from Beijing Pureland Gene Technology Co., Ltd (Beijing, China). Female New Zealand rabbits weighing 2.2 kg was obtained from Guangdong Medical Experimental Animal Center (Guangdong, China) and maintained under standard conditions with a 12-h light/dark cycle. All procedures used in the animal study were approved by the Animal Ethical and Welfare Committee of Sun Yat-sen University (Approval No. SYSU-IACUC-2023-B0929) and followed the National Institutes of Health guidelines for laboratory animal care and use.

### Fabrication of PBIMNP

The paper battery for powering the PBIMNP was purchased from Jiangsu Enforsai Flexible Electronics Co., Ltd (Changzhou, China). The paper battery has an initial voltage of 1.5 V, an initial capacitance of 20 mAh, and a rated pulse current of 10 mA (5 ms). A flexible PCB for the iontophoresis-driven drug delivery was fabricated from Shenzhen Senmi Zhizhuo circuit Technology Co., Ltd (Shenzhen, China). The MN was fabricated by micro-milling technology from aluminum alloy. The morphology of MN and TA was observed using a field emission scanning electron microscope (SEM) (JSM-6380LA, JEOL, Japan). The temperature-sensitive gelatin was prepared for drug encapsulation. 0.176 g gelatin was dissolved in 1 ml of saline and subsequently incubated at room temperature for 30 min. The swollen gelatin was incubated at 70 °C for 5 min and stirred until fully dissolved. Subsequently, 1 mg/ml TA powder was added and shaken for 60 s. A 200 *μ*l mixture was refrigerated at 4 °C for 3 min to allow the formation of a drug-loaded gelatin matrix. The PBIMNP includes three modules: drug storage module, MN module and iontophoresis module. The drug storage module consists of three parts: an impermeable ring, an agarose gel, and a reservoir. The reservoir was prepared by the gelatin loaded with TA. The agarose gel and the reservoir were embedded in the impermeable ring. The MN was inserted into the drug storage module. The flexible PCB was connected with the paper battery for iontophoresis.

### Tests of PBIMNP performance

The current density of PBIMNP for iontophoresis on the rat’s dorsal skin was measured using a digital multimeter (DSOX3052T, Agilent, California, USA). The electrical impedance of PBIMNP fixed on the rat’s dorsal skin were recorded using an impedance analyzer (Agilent E4980A LCR Meter, Palo Alto, CA, USA). The PBIMNP was pressed on the skin surface of ear scar for 2 min. After removal of PBIMNP, the recovery process of the poked microchannels on the HS was observed and recorded. The microchannels recovery of normal tissue was also recorded. The temperature changes during the drug administration on the rabbits were captured using an infrared camera (HM-TP7WL-HB, HIKMICRO, Hangzhou, China). To investigate the performance of HS, we employed optical coherence tomography (OCT, HSO-2000, TEK SQRAY, China) and HE to observe its tissue structure. The puncture performance of PBIMNP and fracture performance of MN were tested using a universal material testing machine (Nano 17 Titanium, ATI Industrial Automation, USA) according to our previous reported experimental procedures^41,42^.

### Cell culture and cell proliferation assay

The HSFbs were cultured in DMEM/F-12 medium supplemented with 10% fetal bovine serum at 37 °C. For the CCK-8 assay, HSFbs were seeded in 96-well plates at a density of 5 × 10^3^ cells/well and incubated for 24 h to promote adhesion. Subsequently, the original medium was replaced with 100 μL of fresh medium containing TA, Gelatin, and Gelatin loaded with TA. After further incubation for 24 and 48 h, the absorption at 450 nm of each well was measured using an ELISA kit from the CCK-8 assay to determine cell viability.

After 24 h of immersion in fresh mediums, the MN were exposed to MN-soaked medium by replacing the medium in the 96-well plate. After incubation for 24 and 48 h, the absorption of each well was measured using CCK-8 assay, while cell viability was determined using enzyme-linked immunosorbent assay (ELISA).

### In vitro permeation performance tests

The Franz diffusion cells (TP-3A, Henan Baize Instrument Co., Ltd.) were employed for in vitro penetration tests on HS skin of rabbit ear. Initially, the subcutaneous cartilage of the rabbit ear HS skin was removed and assembled between the donor pool and recipient pool. The recipient pool was filled with PBS and maintained at 37 °C. Subsequently, in vitro permeation tests were divided in four administration groups (*n* = 3), as listed in Table 1: (1) Cream group, HS skin treated with 200 *μ*g TA cream, (2) MNP group, HS skin treated by microneedles patch loaded with 200 *μ*g TA, (3) PBIMNP group, HS skin administrated with PBIMNP loaded with 200 *μ*g TA using MN penetration and 1.5 V iontophoresis,(4) Injection group, HS skin injected with 200 *μ*g TA suspension. The Cream group was evenly applied TA cream on HS surface. The recipient container was sampled at 2, 5, 10, 20, 30, 45, 60, 120, 240, 780, 720, and 1440 min with 1 ml each time. 1 ml of PBS was added after each sampling to maintain consistency. After the test, the HS tissue was collected and gently wiped with a cotton. Subsequently, 2 ml of methanol was added for lysing and grinding in a mortar. The resulting supernatant after centrifugation represented the TA concentration in HS tissue. The samples were filtered through a microporous filter membrane (pore size of 0.22 *μ*m) and then tested using a HPLC analysis (LC-20AT binary pump, Shimadzu, Kyoto, Japan). The content level for target TA was monitored specifically at 240 nm.

**Table 1:** Administration groups for in vitro permeation tests

Groups	Schematic illustration	Drug	Penetration	Iontophoresis	TA dosage
Cream		TA Cream	–	–	200 *μ*g
MNP		Gelatin + TA	MN	–	200 *μ*g
PBIMNP		Gelatin + TA	MN	1.5 V	200 *μ*g
Injection		TA suspension	Intradermal injection	–	200 *μ*g

### In vivo drug administration tests

#### Establishment of HS models

The New Zealand rabbits were anesthetized via the ear marginal vein using pentobarbital sodium (1%, 30 mg/kg). Three wounds with a diameter of 10 mm were created in the auricular abdominal region, with excision of the subcutaneous periosteum while preserving the cartilaginous structure. Subsequently, the wounds were disinfected with povidone-iodine for 7 days. On the 7th day, saline was used to soften the wound and remove scabs for promoting wound healing. Finally, the rabbit ear scar models were created at the 30th day.

#### Pharmacodynamics studies

The HS model were randomly divided into 7 groups, as listed in Table 2: (1) NS group, normal skin without any treatment, (2) HS group, HS skin without any treatment, (3) Cream group, HS skin treated with 400 *μ*g TA cream, (4) MN group, HS skin penetrated with MN, (5) MNP group, HS skin treated by MNP loaded with 400 *μ*g TA, (6) PBIMNP group, HS skin administrated by PBIMNP loaded with 400 *μ*g TA using MN penetration and 1.5 V iontophoresis for 30 min, and (7) Injection group, HS skin injected with 400 *μ*g TA suspension. The rabbits had a total of 6 wounds, with 3 wounds on each ear. The normal skin of the rabbit ear was employed as the control. The administration of each cycle lasts for 7 days, and a total of 3 cycles were conducted. Twelve female New Zealand rabbits weighing approximately 2.2 kg were selected and randomly divided into 4 groups (*n* = 3). Each group represented a administration cycle. Subsequently, the HS tissue was photographed and measured, and histological and biological tests were conducted on day 37 (7 days after the 1st administration), day 44 (7 days after the 2nd administration), and day 51 (7 days after the 3rd administration) following establishment of the HS model on day 30. After the HS model was established (day 30), 7 days after the 1st administration (day 37), 7 days after the 2nd administration (day 44) and 7 days after the 3rd administration (day 51), the HS tissues were photographed and measured for histological and biological tests.

**Table 2:** In vivo administration groups

Groups	Schematic illustration	Drug	Penetration	Iontophoresis	TA dosage
NS		–	–	–	–
HS		–	–	–	–
Cream		TA Cream	–	–	400 *μ*g
MN		–	MN	–	
MNP		Gelatin + TA	MN	–	400 *μ*g
PBIMNP		Gelatin + TA	MN	1.5 V,30 min	400 *μ*g
Injection		TA suspension	Intradermal injection	–	400 *μ*g

The appearance of HS tissue was recorded using a macro-camera. Following a 2 s pressure on the HS tissue with a slide, the changes in HS were observed and evaluated based on the Vancouver Scar Scale (VSS). The brightness and color changes of HS tissue were quantify using Image J. Additionally, the thickness of HS tissue was measured using a Vernier caliper and converted into a scar index (SEI). Furthermore, the hardness of HS tissue was assessed using a Shore hardness tester (Shore HT-6510OO, Guangzhou Landtek Instrument Co., Ltd, China).

#### Histological assays

The HS tissues were wrapped in tin foil and fixed in 4% paraformaldehyde for 48 h, then embedded in paraffin and sectioned. The embedded sections underwent histological staining with HE, Masson’s trichrome, and Sirius red, respectively. The HE was employed to visualize tissue distribution, quantify somatic cell density per unit area, and assess inflammation. The Masson’s trichrome was utilized to evaluate collagen volume fraction (CVF) within the tissues. The HE and Masson sections were observed using EVOS FL Auto microscope (Life Technologies Co., Ltd, USA) and analyzed with Image J. The Sirius red staining was employed to determine the ratio of type I to type III collagen proteins in the tissues. The stained sections with Sirius red staining were observed using NIKON digital sight DS-FI2 camera (Nikon Instrument Co., Ltd, Japan) and analyzed with Image-Pro Plus 6.0 software to calculate the collagen protein ratio.

#### Total RNA isolation and real-time qPCR (RT-qPCR)

Total RNA was isolated from the collected samples using TRIzol reagent. After treatment with DNase I, the RNA was reverse transcribed to cDNA using the agarose gel electrophoresis. The same amount of cDNA was used for RT-qPCR. Cycles were performed at 95 °C for 3 min followed by 40 cycles of 95 °C for 5 s and 60 °C for 30 s. The fold change was calculated using the 2^−ΔΔ*CT*^ method. The oligonucleotide primers used for PCR amplification were as follows:

GAPDH:

Forward primer: ACC ATC TTC CAG GAG CGA GAT

Reverse primer: TGA TGA CCC TTT TGG CTC CG

TGF-*β*1:

Forward primer: GTG GAC ATC AAC GGG ATC AG

Reverse primer: GCA GTT CTT CTC TGT GGA GC

Collagen I:

Forward primer: CCA GAG TGG AGC AGT GGT TAC

Reverse primer: TGC AGG TTT CGC CAG TAG AG

#### Western blotting assay

The skin tissues were digested and grounded with presselly 24 (Bertin, France) in RIPA lysate and total proteins were extracted with a mixture of protease inhibitors. The total protein concentration was then detected with BCA protein detection kit. Skin tissue bundle proteins were isolated by 10% sodium dodecyl sulfate polyacrylamide gel electrophoresis and transferred to PVDF membrane, which was sealed with 5% emulsion at room temperature. The proteins and primary antibodies were incubated at 4 °C overnight. The PVDF membrane was washed with 0.1% Tween PBS (PBST), the secondary antibodies were incubated at room temperature for 2 h, and the PVDF membrane was washed with PBST again. The protein signals were detected by an automated chemiluminescence image analysis system and gray analysis was performed using Image J.

#### Statistical analysis

The data were presented as mean ± standard deviation. Statistical comparisons were performed using one-way analysis of variance (ANOVA) after normality and homogeneity tests using SPSS (version 26.0, International Business Machines Corporation, New York, USA). The Turkey test was used for post analysis of individual groups, with *p* < 0.05 considered statistically significant.

## Conclusion

In this study, we developed a paper battery powered iontophoresis-driven microneedles patch for HS treatment with a delivery strategy that involves “pressing and poking, phase transformation, and diffusion and iontophoresis”. The microchannels in HS created by PBIMNP puncture were lasted for 420 min without closure (30 min for normal skin), thereby significantly enhances the efficiency of transdermal drug delivery into HS tissues. The storage and controlled release of TA powder was accomplished through the phase transformation of gelatin. The iontophoresis-driven drug delivery of PBIMNP was powered using a thin paper battery, achieving high integration. In vitro experiments demonstrated that PBIMNP could deliver 90.19% of the drug effectively into HS tissue and retained 51.78% of the drug in HS tissue, showing excellent delivery efficiency. In vivo administration of rabbit ear scars also demonstrated the significant efficacy of PBIMNP in the HS management. Notably, the PBIMNP effectively avoid the side effects associated with injection, reduced the mRNA and proteins expression of HS, and facilitated a regular arrangement of collagen fibers. Therefore, the highly integrated PBIMNP provides a possibility for home-care management of HS.

## Supplementary information

Supplementary Material

Video S1

Video S2

Video S3
