# Supplementary material for: Paper battery powered iontophoresis microneedles patch for hypertrophic scar treatment
Source: Microsyst Nanoeng. 2025 Mar 10;11:46. doi: 10.1038/s41378-024-00823-0 (PMC11894194; doi:10.1038/s41378-024-00823-0)
Supplement: Supplementary file 1 — Supplementary Material [file 41378_2024_823_MOESM1_ESM.docx]

***Supplementary Material***

**Paper battery powered** **iontophoresis microneedles patch for hypertrophic scar treatment**

Jie Gao^1^, Fuqian Chen^1^, Chen Wang^1^, Jingbo Yang ^1^, Ying Zheng^1^, Bin Liu^1^, Gang Nie^2^, Linyu Zhu^2, *^, Shuo Wu^1,3, *^, Xi Xie^4^, Lelun Jiang^1, *^

^1^ Guangdong Provincial Key Laboratory of Sensor Technology and Biomedical Instrument, School of Biomedical Engineering, Shenzhen Campus of Sun Yat-Sen University, Shenzhen, 518107 PR China.

^2^ Department of Dermatovenereology, The Seventh Affiliated Hospital, Sun Yat-sen University, Shenzhen, 518107 PR China.

^3^ Department of Otolaryngology, The Third Affiliated Hospital, Sun Yat-Sen University, Guangzhou, 510630 PR China.

^4^ State Key Laboratory of Optoelectronic Materials and Technologies, School of Electronics and Information Technology, Sun Yat-sen University, Guangzhou 510006, China

* The corresponding authors, Tel: +86 20-39332153 E-mail: [jianglel@mail.sysu.edu.cn](mailto:jianglel@mail.sysu.edu.cn); [wush68@mail.sysu.edu.cn](mailto:wush68@mail.sysu.edu.cn); [zhulinyu@sysush.com](mailto:zhulinyu@sysush.com);

**1. Size and performance of different types of microneedles**

Table S1 presents the size and performance of microneedles patches, including drug delivery approach, types of microneedles, and advantages and disadvantages. Based on the drug delivery approach, microneedles patches can be categorized into three types: "Poke," "Dissolve & diffusion," and "Poke, diffusion & iontophoresis". (1) **Poke**: This type of microneedles usually uses drug-free MN, with a diameter of 230-300 μm, a height of 500-1500 μm, and a quantity ranging from 100 to 225. It is simple to use, but has a poor therapeutic effect due to the absence of drugs. Prolonged wear may cause allergic reactions. (2) **Poke, dissolve & diffusion**: This type of microneedles is soluble, with a diameter of 300-450 μm, a height of 500-1200 μm, and a quantity ranging from 100 to 225. Dissolvable microneedles can load drugs. However, the drug load is low and usually single delivery. (3) **Poke, diffusion & iontophoresis**: This type of microneedles usually uses solid metal MN with a diameter of 600 μm, a height of 1000 μm, and a total quantity of 61. Solid metal MN possess better mechanical strength. After iontophoresis, the patches not only enable repeated poke and delivery but also facilitate active administration.

**Table S1** **Size and performance of microneedles patches**

| Delivery approach | Type of MN | Diameter (µm) | Height (µm) | MN Number | Advantages and disadvantages | Ref. |
| --- | --- | --- | --- | --- | --- | --- |
| Poke | Drug free MN | 230- 300 | 500-1500 | 100-225 | Poor therapeutic effect  Cause allergic reactions | 1, 2 |
| Poke, dissolve & diffusion | Dissolv-able MN | 300- 450 | 500-1200 | 100-225 | Low mechanical strength  Low drug loading  Single delivery | 3-7 |
| Poke, diffusion & iontophoresis | Solid metal MN | 600 | 1000 | 61 | Better mechanical strength  Repeat poke and delivery  Active administration | This work |

**2. Design of PBIMNP**

Figure S1 shows the engineering drawing of PBIMNP. Figure s1a shows the overall design of the PBIMNP, with a size of 35 × 40 ×2.1 mm^3^. Figure S1b-S1e shows the key component designs of the PBMNIP. Figure S1b shows the design of the PCB. The external dimensions of the PCB are 35 × 40 mm^2^ and an internal ring with the radiuses of 10 mm and 15 mm, respectively. The PCB dimensions are precisely matched to the paper battery, and the internal shape aligns with the agarose. Figures S1c&S1d show the front view and left view of MN. The MN has a two-stage roundtable design with an inner diameter of 11 mm and an outer diameter of 13 mm. The base diameter and the height of conical microneedles are 600 µm and 1000 µm, respectively. Figure 1e shows the design of the gasket. The gasket size is 35 × 35 mm^2^, and the size of the inner ring is consistent with the agarose.

**
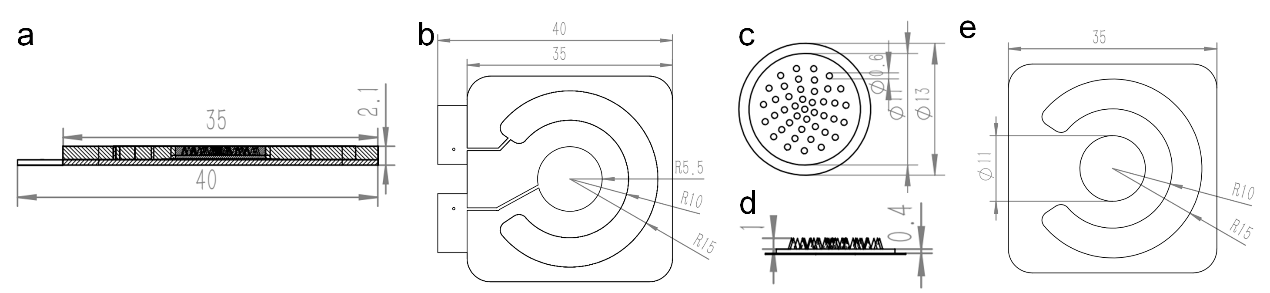
**

Figure S1 Engineering drawing of PBIMNP. (a) Left view of PBIMNP. (b) Design of PCB. (c) Front view of MN. (d) Left view of MN. (e) Front view of gasket.

**3.Phase transformation performance of gelatin**

The storage and release of TA in PBIMNP was achieved through the phase transformation of gelatin. Figure S2 illustrates the dynamic process of gelatin blocks placed on the radial side of human forearm at physiological temperature, where human body temperature-initiated phase transformation at 120 s, followed by gradual deepening until complete transition occurred at 170 s with increasing fluidity observed thereafter (200 s). Video S2 further demonstrates rapid phase transformation of gelatin under physiological conditions (~2-3 min), enabling efficient drug delivery.


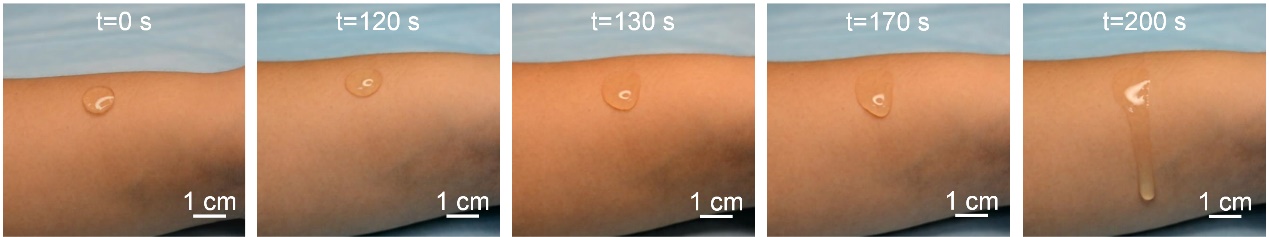


Figure S2 Phase transformation of gelatin on human forearm at 0 s, 120 s, 130 s, 170 s and 200 s, respectively.

**4.** **Characteristic of triamcinolone acetonide**

The drugs are delivered to HS tissues via passive diffusion and active iontophoresis. The efficiency of passive diffusion and active iontophoresis is greatly affected by the microchannels created by PBIMNP and the applied potential, repectivley. Thus, the particle size and Zeta potential of TA were tested, as shown in Figure S3. It was observed that TA particles with an average diameterless than 75 μm accounted for 94.93%, while those less than 187.5 μm constituted 100% (Figure S3a). Notably, the diameter of TA particles was significantly smaller than the microchannel created by PBIMNP (300 μm), suggesting that drug delivery to HS tissues via microchannels was feasible. Figure S3b shows the Zeta potential of TA particles was 3.76 ± 0.25 mV.


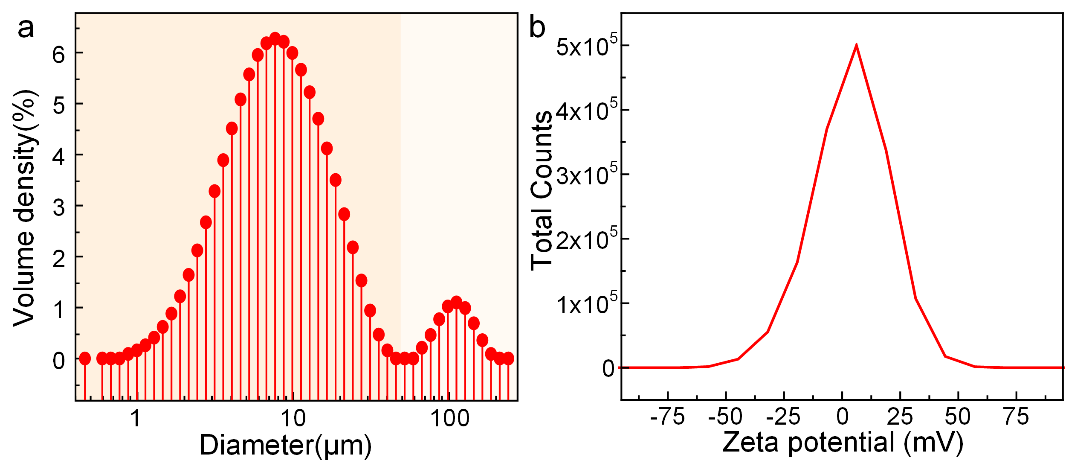


Figure S3 Characterization of TA particles. (a) The diameter distribution of TA particles. (b) The Zeta potential distribution of TA particles.

**5. Characterization of PBIMNP**

Figure S4a is the SEM image of the gelatin. Homogeneous pores can be observed in gelatin which could be used for drug storage. Figure S4b is the optical image of the MN, which owns a sharp tip. Figure S4c is the ultra-depth three-dimensional (3D) image of the MN. The height of a needle is approximately 1000 μm.


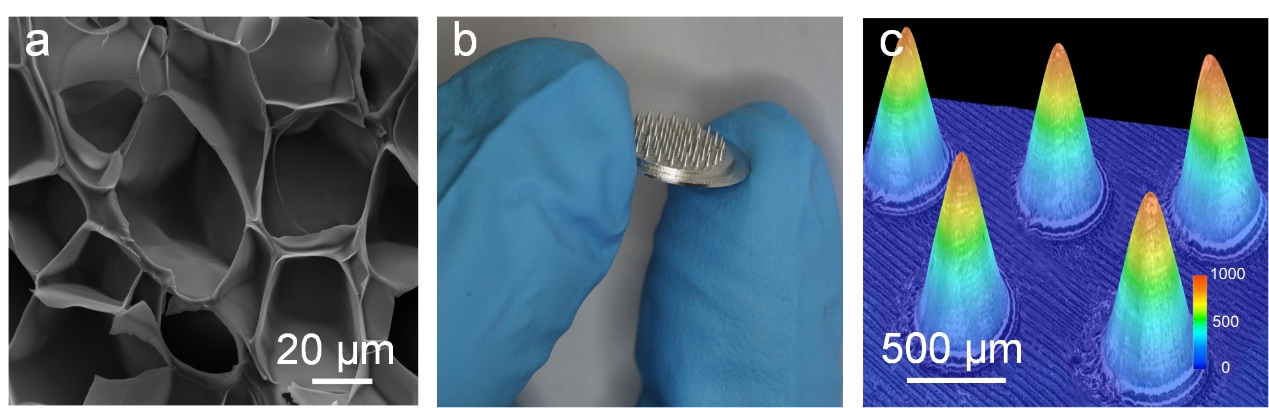


Figure S4 Characterization of PBIMNP. (a) The SEM image of the gelatin. (b) The optical image of MN. (c) The ultra-depth 3D image of MN.

**6. Electrical performance of paper battery**

The impedance of human skin in dry, sweat and wound is approximately 2000 Ω, 1000 Ω and 800 Ω, respectively. The current and voltage output performance of the paper battery were investigated at a dry skin impedance of approximately 2000 Ω, as shown in Figure S5. The paper battery output a stable current and voltage for 60 min (Figure 5a), indicating the reliability of voltage source for iontophoresis. The paper battery output performance of the paper battery under various resistances (500 Ω, 1000 Ω, 1500 Ω, 2000 Ω, 2500 Ω, and 3000 Ω) were further investigated during gelatin phase transformation (Figure S5b). As the resistances increased from 500 Ω to 3000 Ω, the current gradient of the paper battery decreased, while the voltage remained stable, and vice versa. It indicated that the output of the paper battery remained stable even under changing resistances, providing a stable output for the iontophoresis of PBIMNP.


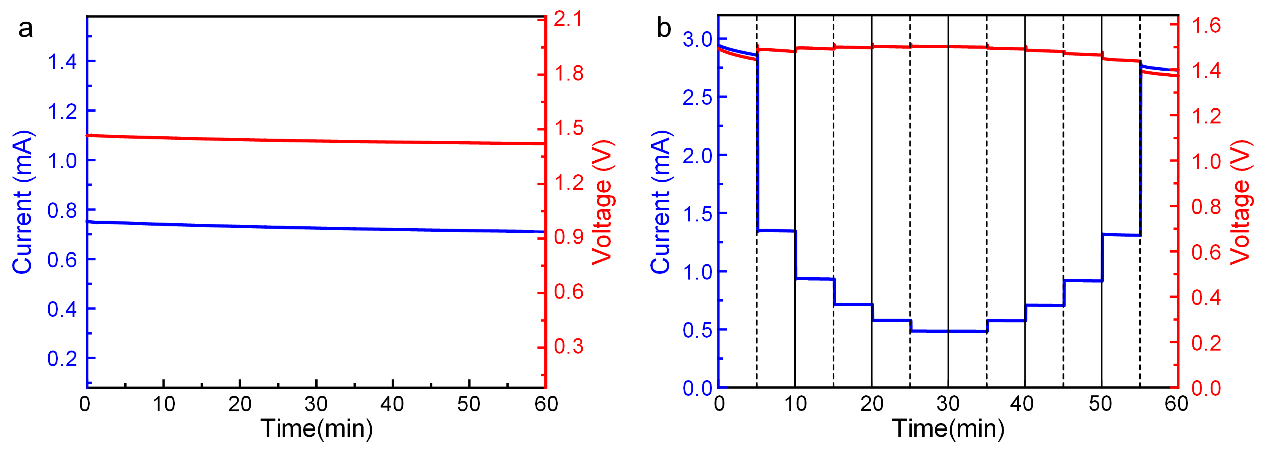
Figure S5 Output performance of the paper battery. (a) Current and voltage performance of paper battery over 60 min at an impedance of 2000 Ω. (b) Current and voltage output performance of paper battery loaded with various resistances.

**7.** **Long-term Stability of PBIMNP**

Figure S6 shows the efficiency of gelatin-wrapped TA after 28 days of storage at 4°C. The efficiency of TA in gelatin was 93.52 ± 1.47% on day 7 and remained at 90.07 ± 2.39% on day 28. It demonstrated that the gelatin-wrapped TA had excellent long-term stability at low temperatures and can be used for HS treatment.


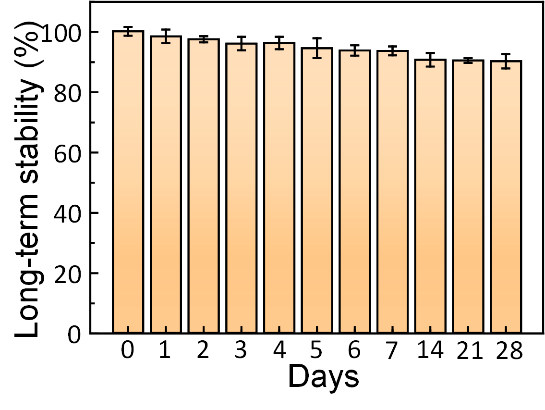


Figure S6 Long-term stability of PBIMNP for 28 days.

**8. Mechanical performance of PBIMNP**

Figures S7a&b show the OCT and HE images of HS tissue after PBIMNP puncture. The depth and diameter of the microchannels were approximately 600 μm and 300 μm, respectively. It indicates that PBIMNP could effectively penetrate dense tissue on HS skin and form uniform microchannels for drug delivery. Due to its high density and hardness, HS puncture required a larger force. The fracture performance of PBIMNP during usage was further investigated to ensure its safety. Figure S7c shows the mechanical performance of PBIMNP when punctured on a steel plate. The load force gradually increased with displacement. The load force reached 3.1 N/needle, which significantly exceeded the penetration force required for HS (1.7 N/needle). It demonstrates that PBIMNP can effectively penetrate the dense HS tissue without fracture, thereby ensuring its safety.


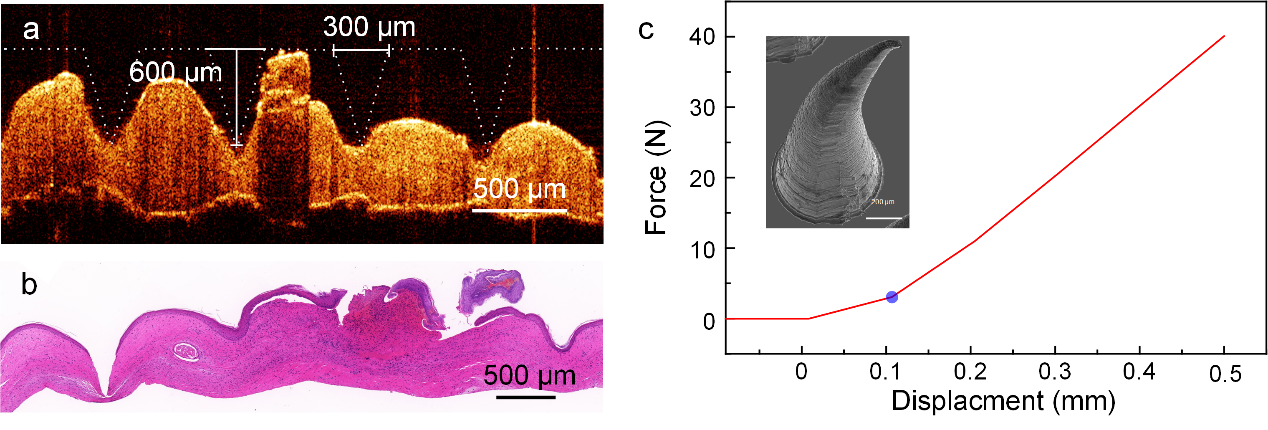


Figure S7 Mechanical performance of PBIMNP. (a) OCT images of HS tissue after PBIMNP puncture. (b) HE images of HS tissue after PBIMNP puncture. (c) Mechanical resistance of PBIMNP when punctured on a steel plate.

**9. Puncture performance of PBIMNP**

Figure S8 shows the microchannels recovery after PBIMNP puncture in HS and normal skin. The microchannels created on HS skin remained visible even after 60 min of recovery, and it took approximately 420 min for complete healing (Figure S8a). In contrast, microchannels in normal skin completely healed within 30 min (Figure S8b). The longer recovery of microchannels in HS tissue may be attributed to its skin tension. The persistent opening of microchannels in HS skin holds promising implications for long-term iontophoresis-driven drug delivery, offering high potential for HS treatment.

**
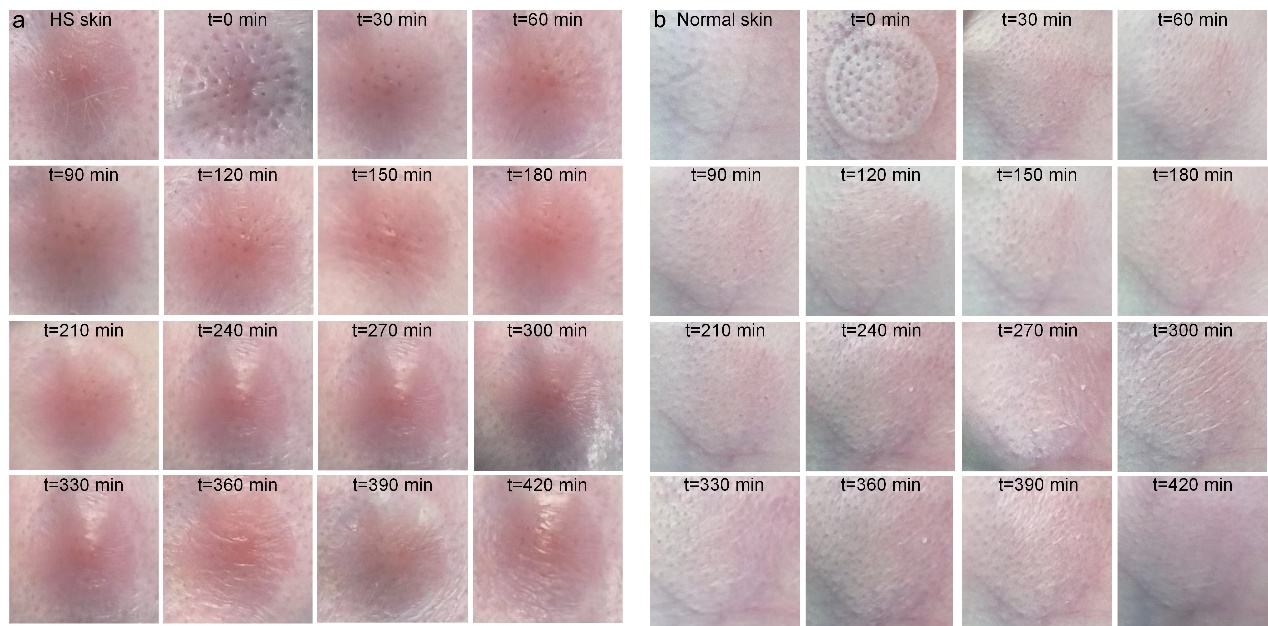
**

Figure S8 Puncture performance of PBIMNP: (a) The recovery process of HS skin before and after puncture. (b) The recovery process of normal skin before and after puncture.

**10.** **Diffusion behavior of drugs in skin**

Figure S9 shows the distribution of Rhodamine B within the skin under three different administration approaches: free diffusion, MNP, and PBIMNP. Figure S9a shows the fluorescence images of the skin treated with free diffusion. The fluorescence intensity gradually decreases with depth, indicating its limited diffusion. Figure S9b shows the fluorescence images of the skin after MNP puncture. The fluorescence pattern aligns closely with MN arrangement, indicating successful puncture and effect drug distribution within the tissue. Compared with free diffusion approach, MNP significantly increased the fluorescence intensity, indicating the promoted diffusion in deeper tissue. Figure S9c shows the fluorescence image of the skin after PBIMNP puncture. Compared to the MNP group, iontophoresis significantly enhances both fluorescence intensity and diffusion depth, indicating its effectiveness in deep tissue diffusion. The fluorescence intensity was quantitatively analyzed with Image J, as shown in Figure S9d. Both MNP and PBIMNP could enhance drug diffusion efficiency in the skin, with the PBIMNP group demonstrating the most significant effect, indicating its superior potential for deeper drug diffusion.

**
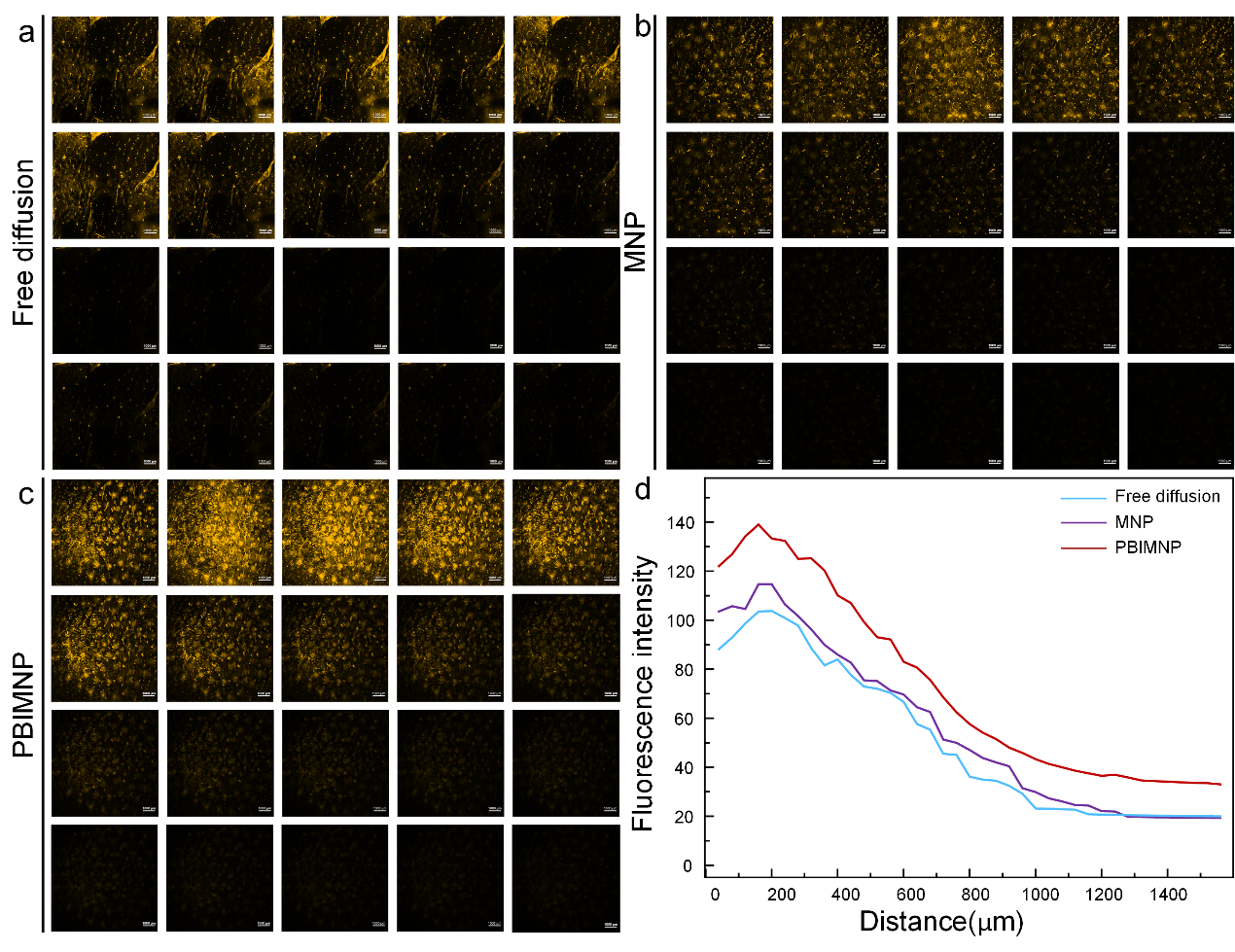
**

Figure S9 Diffusion and fluorescence intensity distribution within the skin. (a) The fluorescence images of the skin treated with free diffusion. (b) The fluorescence images of the skin administrated with MNP. (c) The fluorescence images of the skin administrated with PBIMNP. (d) Quantitative analysis of fluorescence intensity with distance of different administration approaches.

**11.** **Flow field distribution of drugs in the skin**

Figure S10 shows the simulated drug diffusion distribution within the skin of four administration approaches. In Cream group, the flow field is mainly concentrated around the skin surface with lower concentrations. The MNP group exhibits a more focused flow field, with significantly increased penetration. The PBIMNP group shows a deeper diffusion depth, with the flow field primarily concentrated within the skin, indicating that the drug mainly diffuses into the tissue. In Injection group, the flow field rapidly spreads outward through the injection site. Overall, the flow field distribution shows that the PBIMNP group is primarily concentrated within the tissue, indicating a more extensive drug administration within the tissue.

**
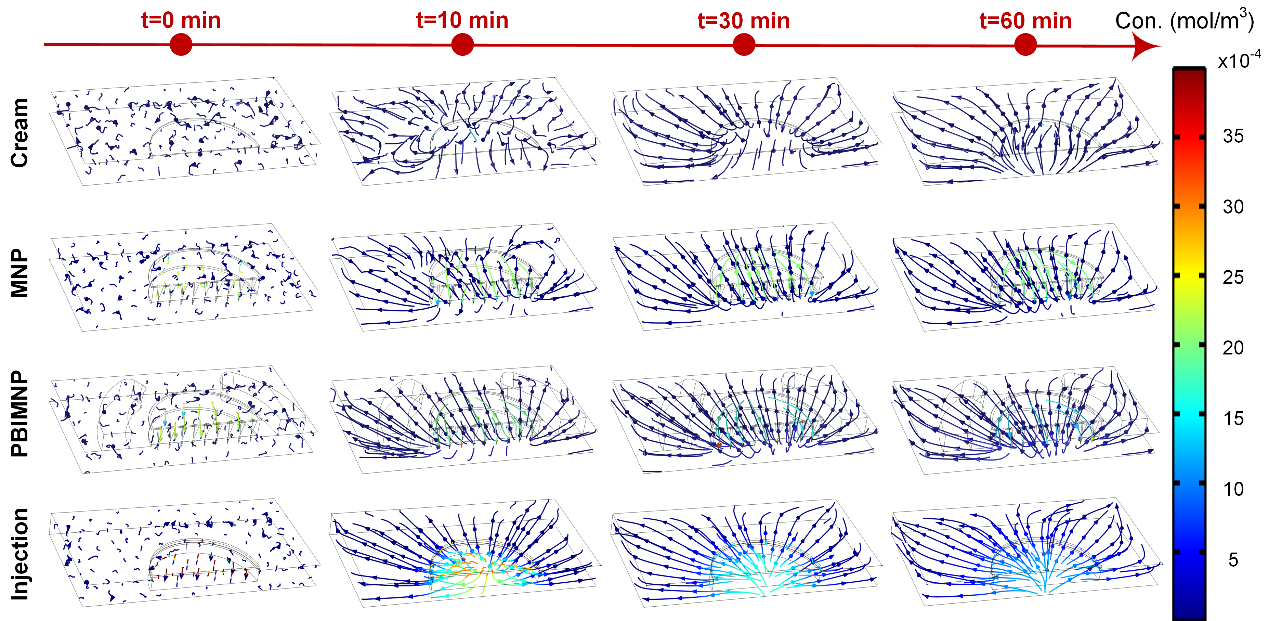
**

Figure S10 The flow field drug diffusion distribution within the skin of four administration approaches.

**11. *In vivo* experiments of PBIMNP**

Figure S11 shows the morphology of HS skin before and after administration. A uniform wound was created on the skin of the rabbit ear through surgical (day 1), leading to a formation of HS after 30 days due to the self-repair of the skin. The skin surface exhibited a raised and dark red color, which is indicative of HS formation. After 3 administrations, the scar swelling and raised were significantly subsided in the MNP, PBIMNP, and Injection group. In contrast, the HS and Cream group still exhibited tightness, darkness in coloration, and raised condition. Furthermore, compared with the HIIIMNP group, the Injection group had a concave-convex surface, potentially attributed to the uneven distribution of drugs in HS tissue caused by injection. It suggests that PBIMNP enables painless and uniform drug delivery in HS tissue, offering a promising therapeutic approach for HS.


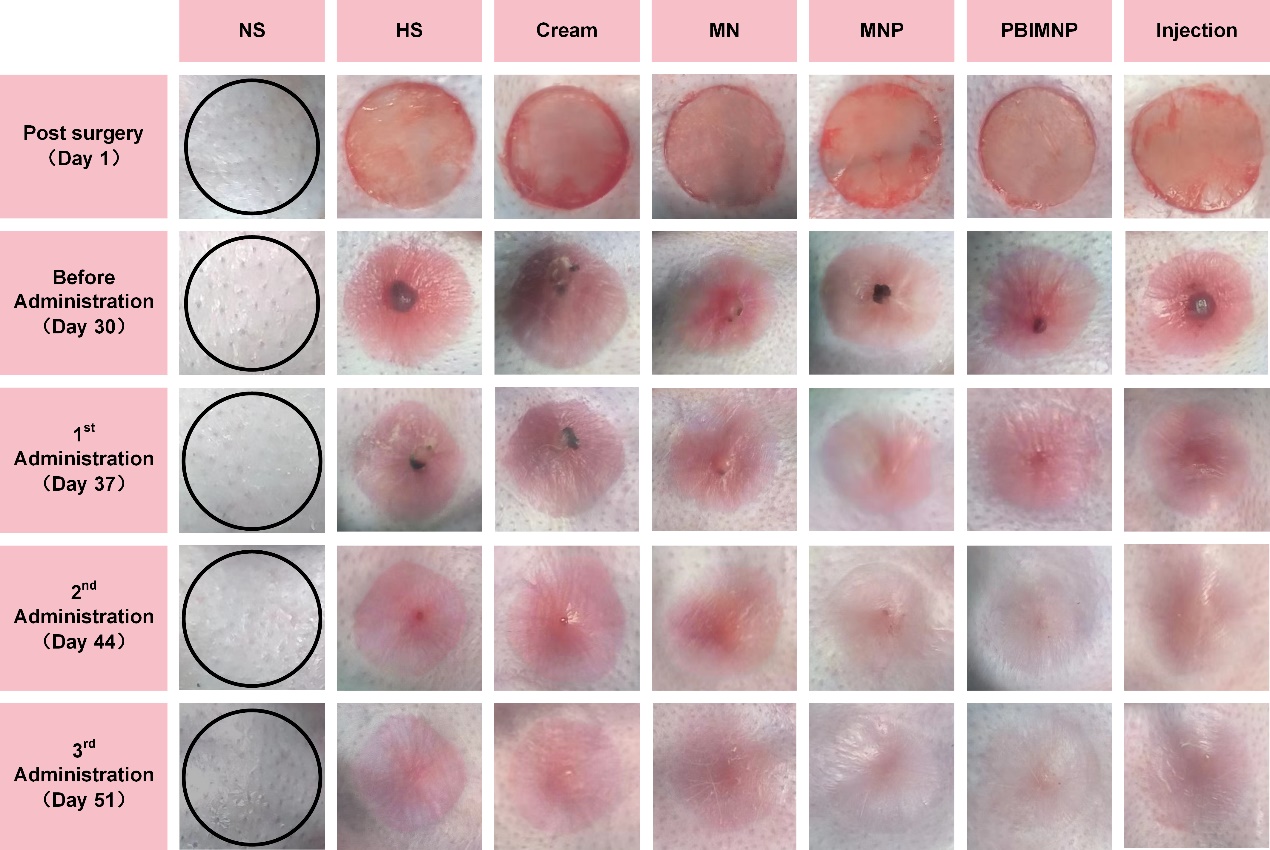


Figure S11 Morphology images of HS skin before and after administration.

**12. Histological Study**

Figure S12a shows HE images of HS skin before and after administration. On day 30, the normal tissue exhibited regular arrangement and uniform tissue spacing. In contrast, other groups displayed a dense and disordered tissue structure, indicating successful establishment of HS model. After 3 administrations, HS and Cream still exhibited a dense tissue, whereas MN group displayed numerous fine fibers arranged evenly with a relatively compact structure. Conversely, MNP, HIIIMNP, and Injection group showed abundant coarse fibers arranged regularly similar to normal tissue with a relatively loose tissue structure. It suggests that PBIMNP can achieve the therapeutic effect on HS by regulating the distribution of collagen fibers in HS tissues. The somatic cell count in HS tissue was further quantified, as shown in Figure S12b. The somatic cell count in HS tissue of PBIMNP group was 1666 ± 23/mm^3^ on day 30 and 838 ± 30/mm3 on day 51, and the normal tissue was 786 ± 7.94/mm^3^, indicating a significant reduction.


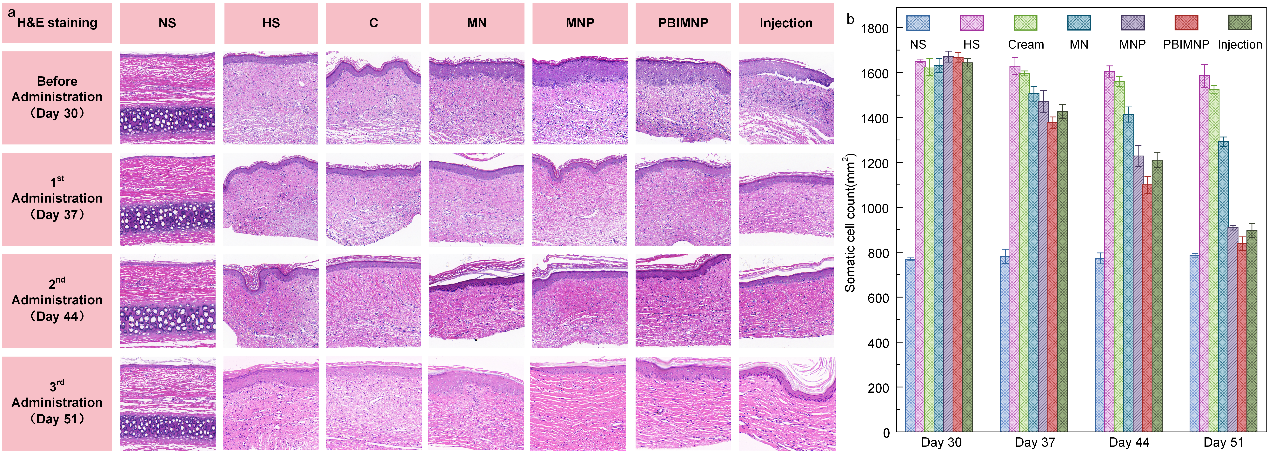


Figure S12 Histological analysis of HS tissue before and after various administrations. (a) HE images of HS tissue before and after administration. (b) Somatic cell counts of HS tissue after HE staining.

1.Yeo, D. C., Balmayor, E. R., Schantz, J.-T. & Xu, C. Microneedle physical contact as a therapeutic for abnormal scars. European Journal of Medical Research 22 (2017).

2. Zhang, Q. et al. Down-regulating scar formation by microneedles directly via a mechanical communication pathway. ACS nano 16, 10163-10178 (2022).

3. Yang, B., Dong, Y., Shen, Y., Hou, A. & Wu, C. Bilayer dissolving microneedle array containing 5-fluorouracil and triamcinolone with biphasic release profile for hypertrophic scar therapy. Bioactive Materials 6, 2400-2411 (2021).

4. A, S. L. et al. Strategy for hypertrophic scar therapy: Improved delivery of triamcinolone acetonide using mechanically robust tip-concentrated dissolving microneedle array - ScienceDirect. Journal of Controlled Release 306, 69-82 (2019).

5. Zhang, N. et al. Co-delivery of triamcinolone acetonide and verapamil for synergistic treatment of hypertrophic scars via carboxymethyl chitosan and Bletilla striata polysaccharide-based microneedles. Carbohydrate polymers 284, 119219 (2022).

6. Ning, X., Wiraja, C., Chew, W. T. S., Fan, C. & Xu, C. Transdermal delivery of Chinese herbal medicine extract using dissolvable microneedles for hypertrophic scar treatment. Acta Pharmaceutica Sinica B 11, 2937-2944 (2021).

7. Hao, R. et al. Transdermal delivery of Protocatechuic aldehyde using hyaluronic acid/gelatin-based microneedles for the prevention and treatment of hypertrophic scars. European Journal of Pharmaceutics and Biopharmaceutics 184, 202-213 (2023).
